# High-solids ethanol production from waste paper through consolidated bioprocessing using advanced *Saccharomyces cerevisiae* strains

**DOI:** 10.1007/s00449-026-03416-5

**Published:** 2026-08-27

**Authors:** Juliana E. Naudé, Daneal C. S. Rorke, Johann F. Görgens, Eugéne van Rensburg

**Affiliations:** Department of Chemical Engineering, Stellenbosch University, Private Bag X1, Matieland, 7602 South Africa

**Keywords:** Waste paper, Ethanol, High solids fermentation, Consolidated bioprocessing yeast

## Abstract

High-solids simultaneous saccharification and co-fermentation (SScF) of paper wastes were compared in lab-scale fed-batch (vertical) and batch (horizontal) bioreactor cultures. Fermentation of untreated label backing (LB), multilayer paper-based packaging (ML) and potato paper sacks (PS) paper wastes were optimised for solid loading to minimise the exogenous enzyme dosage with advanced consolidated bioprocessing (CBP) yeast strains, to achieve maximum technical performances in terms of ethanol concentration, yield and enzyme efficiency. Using cellulase-producing CBP yeast (Cellusec^®^ 2.0, 3.3 or 4.0) improved the enzyme efficiency by 1.5-fold at a final solid loading of 20% (w/w dry) compared to a higher solid loading of 30% (w/w) in fed-batch SScF. At 20% (w/w) solid loading and 2 FPU/g ds, enzyme efficiencies of 0.109, 0.078 and 0.067 g ethanol/FPU were recorded with corresponding ethanol titres of 59.3, 45.2 and 38.2 g/L and ethanol yields of 58.5, 43.9 and 57.1% of the theoretical maximum for LB, PS and ML, respectively. Whereas batch SScF at 30% (w/w) solid loading and 2 FPU/g ds achieved higher ethanol titres of 65.6, 50.7 and 54.3 g/L, the corresponding ethanol yields and enzyme efficiencies were markedly lower at 36.7, 28.0 and 52.1% of the theoretical maximum and 0.069, 0.050 and 0.062 g ethanol/FPU for LB, PS and ML, respectively. Fed-batch SScF in a vertical bioreactor at 20% (w/w) final solid loading was, therefore, the preferred configuration due to more favourable enzyme efficiencies, ethanol yields and enzyme cost.

**Supplementary Information:**

The online version contains supplementary material available at https://doi.org/10.1007/s00449-026-03416-5.

## Introduction

The global energy demand increased by 2.2% in 2024, which exceeded the average rate of 1.3% per year over the last 10 years [1]. Of this demand, the global use of biofuels for transport consisted of approximately 5 EJ in 2024 [2]. This increase in demand for biofuels, with a concomitant decrease in reliance on fossil fuels was driven by support for gasoline blending and increased transport fuel consumption from renewable sources [1]. While the increase in demand for bioethanol can be attributed to energy for transport, this high-value commodity can also be used as a platform chemical in the pharmaceutical, cosmetic or beverage industry [3]. Waste-based ethanol could alleviate the demand for bioethanol, with advantages such as decreased GHG emissions compared to conventional fossil fuels [4, 5], use of non-edible food crops [6] and mitigating pollution and reduction of waste, such as food waste [7] and paper waste [8].

The use of waste paper for ethanol production is attractive as it is an abundant resource, evident from global consumption of paper and paperboard that reached approximately 419 million metric tons in 2022 [9]. Additionally, as waste paper consists of lignocellulosic material, it is classified as a 2nd generation (2G) ethanol feedstock [8] and the use of 2G ethanol greatly reduces GHG emissions compared to conventional fossil fuels [5] and 1st generation (1G) ethanol [8]. Furthermore, previous studies have determined that waste paper streams, such as office paper, newspaper and cardboard, could be a viable feedstock for microbial conversion into ethanol [10–21]: however, fermentation and anaerobic digestion of unrecyclable streams such as label backing paper (LB), multilayer paper-based packaging (ML) and potato paper sacks (PS) have not yet been investigated.

Although recycling of waste paper into fibrous products is preferred [22, 23], waste paper streams such as LB, ML and PS, which are deemed unrecyclable are often incinerated or landfilled [24]. These disposal methods contribute to the contamination of underground water and air pollution from the release of GHGs and spontaneous landfill fires [21, 25–28]. Therefore, beneficiating unrecycled waste paper to ethanol assists in diverting paper products from incineration and landfill, of which 30–50% are landfilled along with municipal solid waste [21, 25, 29–31]. Moreover, compared to other 2G ethanol feedstocks such as corn stover and sugarcane bagasse that require energy-intensive thermophysical pretreatment [5, 32], some waste paper types including, LB, ML and PS, require little to no energy-intensive thermophysical pretreatment to disrupt the crystalline lignocellulosic structure. The amenability of these materials can be attributed to pulping during paper production, which partially delignifies and disrupts the lignocellulose structure [17, 33–35].

The technical viability of converting waste paper into ethanol is determined by the achievable ethanol concentration (final titre), yield and volumetric productivity under fermentation conditions that would be acceptable for industrial implementation. Typical variables that are key to successful conversion include enzyme dosage (cost), yeast strain (robustness and cellulase secretion), and solid loading (to maximise available carbon without inhibiting mass transfer). For an ethanol fermentation process to be technically viable and industrially attractive, ethanol concentrations greater than 40 g/L are critical due to the energy requirements for downstream ethanol distillation [36–39]. Consequently, a dry solid loading of at least 15–20% (w/w), which is classified as a high-solids fermentation [40–42], is required in a SScF process to achieve the desired final ethanol titre, depending on the lignocellulosic substrate composition and its carbohydrate content [43, 44]. Additionally, operating at high solid loadings include advantages such as reduced overall process costs due to smaller size of processing vessels required per unit of bioethanol produced [38, 39, 42]. However, during batch fermentation, high solid loadings often result in heating and mass (mixing) transfer limitations from viscous slurries [39, 45].

A fed-batch approach to high-solids processes can mitigate heat and mass (mixing) transfer limitations, as observed in several previous studies that reported ethanol concentrations greater than 40 g/L [17, 21, 46, 47]. Furthermore, high-solids fermentations in horizontal rotating bioreactors may mitigate these limitations due to the free fall motion of the particles at low speed mixing [43], compared to high-solids fermentation processes in vertical bioreactors, which often impede heat and mass transfer [39, 45, 48]. High-solids fermentation in horizontal bioreactors has been applied to produce enzymes, single-cell proteins and alcoholic beverages made from rice [40]. This fermentation approach was also successfully applied to ethanol fermentation of paper sludge [12], warranting an investigation into its application in the high-solids fermentation of unrecyclable LB, ML and PS, which to date, has not yet been conducted. The use of a horizontal rotating reactor holds significant value as to date these reactors have not often been used for waste paper fermentation, except for paper sludge [12] and municipal solid waste [49].

The heat and mass (mixing) transfer limitations of high-solids processes can be resolved by implementing higher enzyme dosages for improved liquefaction [13, 50]. However, the associated costs of cellulolytic enzymes can reduce the economic viability of cellulosic ethanol production [8, 13, 51–53]. Therefore, it is pertinent to minimise the exogenous enzyme requirement and associated process costs while simultaneously maintaining or improving ethanol titres and yields. The key to minimising exogenous enzyme dosage lies in combining a simultaneous saccharification and co-fermentation (SScF) process with an advanced recombinant *Saccharomyces cerevisiae* strain engineered for consolidated bioprocessing (CBP), that produces a substantial portion of the enzymes required for hydrolysis, while simultaneously fermenting glucose and xylose into ethanol. In a previous study [35], the use of a CBP strain of *S. cerevisiae*, capable of co-fermenting xylose and glucose and secreting cellulolytic enzymes, resulted in a 2-to 3-fold exogenous enzyme reduction compared to a conventional industrial *S. cerevisiae* yeast strain. Furthermore, in that previous study [35], simultaneous saccharification and fermentation (SSF) was determined to be the preferred process configuration for LB, ML and PS valorisation. Thus, SScF, which efficiently utilises enzyme-producing CBP yeast to minimise the required dosage of external enzymes, which is a substantial processing cost, will be implemented.

This present study aimed to investigate the high-solids fed-batch SScF process configuration in a vertical reactor and the high-solids batch SScF process configuration in a horizontal reactor to assess which would be more suitable for converting the LB, ML and PS waste paper streams to ethanol through CBP. The conditions selected to assess these streams were based on findings in [35]. Three advanced *S. cerevisiae* CBP yeast strains were compared to identify the preferred yeast strain, namely;

- Cellusec^®^ 2.0 that secretes cellobiohydrolase I and endoglucanase, and co-utilises glucose and xylose.
- Cellusec^®^ 3.3 that secretes cellobiohydrolase I, II, endoglucanase and β-glucosidase and co-utilises glucose and xylose.
- Cellusec^®^ 4.0 an improved strain derived from Cellusec^®^ 3.3, with thermotolerant (39 °C) capabilities, which was not tested in the previous study [35].

Increased solid loadings and reduced exogenous enzyme dosages, respectively, higher and lower than the ranges investigated in the previous study [35], were investigated in both SScF systems to identify the preferred process conditions where ethanol concentrations exceed 40 g/L and ethanol yield and enzyme efficiency were maximised.

## Materials and methods

### Waste paper feedstock

LB, ML and PS were sourced in the Western Cape province of South Africa with milling, sterilisation and compositions as previously described [35].

### Simultaneous saccharification and co-fermentation (SScF)

#### Preparation of yeast inoculum and fermentation media

Three industrial *S. cerevisiae* strains were used in this study: CBP strains Cellusec^®^ 2.0, Cellusec^®^ 3.3, and Cellusec^®^ 4.0, a thermotolerant strain engineered to tolerate temperatures as high as 39 °C, based on Cellusec^®^ 3.3 (NovelYeast, Leuven-Heverlee, Belgium). The yeast extract-peptone-dextrose (YPD) seed culture media was made and used as stated in [35]. A seed train method was used to make 1.8 mL aliquots of the yeast strains, which were then stored at – 80 °C in 35% (v/v) of an 80% (v/v) glycerol solution as cryoprotectant. The yeast inoculum was prepared by transferring a 1.8 mL aliquot to each 250 mL baffled Erlenmeyer flask containing 125 mL of the seed culture media. The yeast cultures were incubated at 35 °C in an orbital shaker at 120 rpm, targeting a concentration of 2–3 g dry yeast per kilogram of fermentation mass (2–3 g/kg). The Cellusec^®^ 2.0, Cellusec^®^ 3.3 and Cellusec^®^ 4.0 cultures were incubated until the cultures reached mid-exponential growth at 14 h. Less and more nutrient dense fermentation media were prepared as stated in "Fermentation media comparison", the runs conducted for high-solids fed-batch SScF and high-solids batch SScF used a less nutrient dense, corn steep liquor (CSL) fermentation media, favouring a more low-cost medium [54].

#### Fermentation media comparison

The effect of a more nutrient rich medium and a less nutrient rich medium on the ethanol production from the advanced CBP yeast strains was investigated. The less nutrient rich fermentation medium [12, 54, 55] contained 3 g/L CSL (Sigma-Aldrich, South Africa) and 0.62 g/L MgSO_4_·7H_2_O (Kimix, South Africa) and the more nutrient dense yeast extract-peptone (YP) medium contained 20 g/L peptone (Kimix, South Africa) and 10 g/L yeast extract (Merck, Germany).

#### High-solids fed-batch SScF in a vertical reactor

High-solids fed-batch SScF runs were conducted for 144 h in an orbital shaker incubator at 120–150 rpm and 37 °C. All shake flask fermentation runs were conducted in triplicate at a working mass of 100 g in 250 mL baffled Erlenmeyer flasks, fitted with rubber stoppers and S-shaped bubble traps. The fermentation methods used for the SScF approach were adapted from [55–57]. The loss of ethanol to the gas phase was assumed to be negligible, as precautions were taken during feeding and sampling, but could possibly account for slight variation in the ethanol concentrations between the fermentation approaches.

Two final dry solid loadings of 20% and 30% (w/w) were investigated for the three waste paper streams, i.e. LB, ML, and PS. Three enzyme dosages of 2, 3.5 and 5 FPU/g dry solids (ds) with Cellic^®^ CTec3-HS (Novozymes, Denmark) were used to investigate the fermentation performance during exogenous enzyme reduction. The activity of Cellic^®^ CTec3-HS was determined to be 127 filter paper units (FPU)/mL.

Cellusec^®^ 2.0, Cellusec^®^ 3.3 and Cellusec^®^ 4.0 were prepared as described in "Preparation of yeast inoculum and fermentation media". To obtain the target dry yeast concentration of 2–3 g/kg after inoculation, a yeast cream consisting of a higher cell concentration was made by centrifuging the yeast inoculum at 4 500 rpm. The settled yeast pellets were resuspended in the respective yeast cultures’ supernatants ensuring a 10% (v/w) inoculum, so that a portion of the cellulolytic enzymes secreted by Cellusec^®^ 2.0, Cellusec^®^ 3.3 or Cellusec^®^ 4.0 was added to the fermentation broth. The pH was monitored during the runs and remained between pH 4 and 6 for all substrates. The initial dry solids at 0 h in the fermentation broth were 5% (w/w) and feedings of 5% (w/w) were conducted at 12 h, 24 h and then every 24 h until the final dry solid loading was obtained. Samples were taken at 0 h, 6 h, 12 h, 24 h and then every 24 h, directly before waste paper was fed.

#### High-solids batch SScF in a horizontal reactor

High-solids batch SScF runs were performed using 3 L glass jars (referred to as roller bottles) at a working mass of 380 g for 144 h in a horizontal roller incubator at 13 rpm and 37 °C. Two final dry solid loadings of 30% and 40% (w/w) were investigated for LB, ML, and PS. Three enzyme dosages of 2, 3.5 and 5 FPU/g ds with Cellic^®^ CTec3-HS were also considered. Cellusec^®^ 2.0, Cellusec^®^ 3.3 and Cellusec^®^ 4.0 were prepared as described in "Preparation of yeast inoculum and fermentation media". Each roller bottle required 500 mL of the yeast inoculum, which was centrifuged at 4 500 rpm to obtain the target dry yeast concentration of 2–3 g/kg. The settled yeast pellets were resuspended in the respective yeast cultures’ supernatants, ensuring a portion of the cellulolytic enzymes secreted by Cellusec^®^ 2.0, Cellusec^®^ 3.3 or Cellusec^®^ 4.0 was added to the fermentation broth. The pH was monitored during the runs and remained between pH 4 and 6 for all substrates. Samples were taken as described in "High-solids fed-batch SScF in a vertical reactor".

### Analytical methods

The analytical methods followed during this study are described in detail in a previous study and included HPLC preparations and HPLC analysis for glucose, xylose, ethanol, acetic acid and lactic acid quantification [35]. The moisture content was measured using a moisture analyser (A&D ML-50, Japan).

#### Statistical analysis

The statistical analyses followed during this study were the same as in the previous study [35]. Runs were conducted in triplicate and the calculation of means and standard deviation, represented by errors bars in figures, were conducted using Microsoft Excel. Statistical analysis and the interaction between factors were determined using Statistica 14.0 software (TIBCO Software, Palo Alto, CA, USA). Additionally, analysis of variance (ANOVA) and Tukey’s post-hoc tests were conducted to determine the significance of certain variables and to provide further insight into the effect of different experimental conditions on the response variables.

### Calculations

#### Theoretical ethanol (g) from glucose and xylose

1 $$\begin{gathered} {\mathrm{Theoretical~ethanol~}}\left( {\mathrm{g}} \right) \hfill \\ \,\,={\mathrm{dry~solid~loading~}}\left( {\mathrm{g}} \right) \times 0.511 \hfill \\ \,\, \times \left[ {\left( {{f_{{\mathrm{Cellulose}}}} \times 1.11} \right)+\left( {{f_{{\mathrm{Hemicellulose}}}} \times 1.14} \right)} \right] \hfill \\ \end{gathered} $$

where f_Cellulose_ and f_Hemicellulose_ are the fractions of cellulose (g/g) and hemicellulose (g/g) in the waste paper stream, respectively, 0.511 is the mass conversion factor of glucose and xylose to ethanol and 1.111 and 1.14 are the mass conversion factors of cellulose to glucose (g_Glucose_/g_Cellulose_) and hemicellulose to xylose (g_Xylose_/g_Hemicellulose_), respectively.

#### Ethanol produced (g)

2 $$\begin{gathered} {\mathrm{Ethanol~produced~}}\left( {\mathrm{g}} \right)={\mathrm{~}}{C_{ethanol}} \hfill \\ \,\, \times {\mathrm{m~}}\left( {\mathrm{g}} \right) \times {\mathrm{moisture~content~}}\left( {{\mathrm{g}}/{\mathrm{g}}} \right) \times 1/\rho \hfill \\ \end{gathered} $$

where C_ethanol_ is the experimental ethanol concentration (g/L) as indicated by HPLC analysis. The m is the estimated mass in the reactor at the time of sampling. The density of the liquid phase, *ρ* was estimated as 1000 g/L.

#### Ethanol yield (% of theoretical maximum)

3 $$ \begin{aligned} & \% \mathrm{oftheoreticalethanol} \\ & \quad ={\text{}}\left( {{\mathrm{ethanolproduced~}}\left( {\mathrm{g}} \right)} \right) \\ & \qquad /\left( {{\mathrm{theoreticalethanol}}\left( {\mathrm{g}} \right)} \right) \times 100 \end{aligned} $$

where ethanol produced (g) is obtained from Eq. 2 at the desired time point with the corresponding solid loading. The solid loading should be incorporated as it changes during fed-batch.

#### Ethanol yield (g_ethanol_/g_carbohydrates_)

4 $$\begin{gathered} {\mathrm{Ethanol~yield}}={\mathrm{~}}\left( {{\mathrm{ethanol~produced~}}\left( {\mathrm{g}} \right)} \right) \hfill \\ \;\;/\left[ {({\mathrm{dry~solid~loading~}}\left( {\mathrm{g}} \right) \times \left( {{f_{{\mathrm{Cellulose}}}}+{f_{{\mathrm{Hemicellulose}}}}} \right)} \right] \hfill \\ \end{gathered} $$

where ethanol produced (g) is obtained from Eq. 2 at the desired time point with the corresponding solid loading. Where f_Cellulose_ and f_Hemicellulose_ are the fractions of cellulose (g/g) and hemicellulose (g/g) in the waste paper stream, respectively.

#### Enzyme efficiency (g_ethanol_/FPU)

5 $$\begin{gathered} {\mathrm{Enzyme~efficiency~}}\left( {{\mathrm{g~ethanol}}/{\mathrm{FPU}}} \right) \hfill \\ \;={\mathrm{ethanol~produced~}}\left( {\mathrm{g}} \right)/\left( {{\mathrm{enzyme~dosage~}}\left( {{\mathrm{FPU}}/{\mathrm{g~ds}}} \right)} \right. \hfill \\ \left. {\; \times {\mathrm{~mass~of~dry~solids~}}\left( {\mathrm{g}} \right)} \right) \hfill \\ \end{gathered} $$

The solid loading should be incorporated as it changes during fed-batch.

#### Enzyme cost ($/L_ethanol_)

6 $$\begin{gathered} {\mathrm{Enzyme~cost~}}\left( {{{\$ }}/{\mathrm{L~ethanol}}} \right) \hfill \\ \;=\left( {{V_{enzyme}} \times {\rho _{enzyme}} \times cos{t_{enzyme}}\left( {{{\$ }}/{\mathrm{kg}}} \right)} \right) \hfill \\ \;/\left( {{\mathrm{ethanol~produced~}}\left( {{\mathrm{kg}}} \right) \times 1/{\rho _{ethanol}}} \right) \hfill \\ \end{gathered} $$

where $$\:{V}_{enzyme}$$ (L) is the volume of enzyme added, $$\:{\rho\:}_{enzyme}$$ is the density of the enzyme calculated as 1.21 kg/L, the ethanol produced (kg) is obtained from Eq. 2 and $$\:{\rho\:}_{ethanol}$$ was assumed to be 0.789 kg/L. The $$\:{cost}_{enzyme}$$ in $ per kg of enzyme of $2.75/kg [58] (assumed to be obtained from off-site production) was assumed from a literature survey of the cost of enzymes [58–61]. It was noted, from literature that the cost of enzymes not only depend on the commercial cellulase price but depends on supplier agreements, whether it is on-site or off-site production and the carbon source used [52, 53, 62, 63].

## Results and discussion

### Ethanol production of engineered *S. cerevisiae* Cellusec^®^ CBP strains using Avicel cellulose compared to using LB and PS

The ethanol production of the Cellusec^®^ 3.3 and 4.0 CBP yeast strains from Avicel^®^ cellulose were compared to the ethanol production from LB and PS that contain cellulose and hemicellulose. The LB and PS waste paper streams were selected since they had the highest and similar quantities of cellulose and hemicellulose for easier comparison to Avicel^®^ cellulose. Avicel^®^ cellulose is an easily digestible substrate that does not contain hemicellulose, which allowed a direct comparison of the fermentation performance of the strains on a pure hexose carbon source. Additionally, for comparison purposes, the quantity of Avicel^®^ cellulose used was matched with the cellulose content of the LB and PS substrates which was about 50.8% − 51.2% (w/w) [35].

**Fig. 1:**
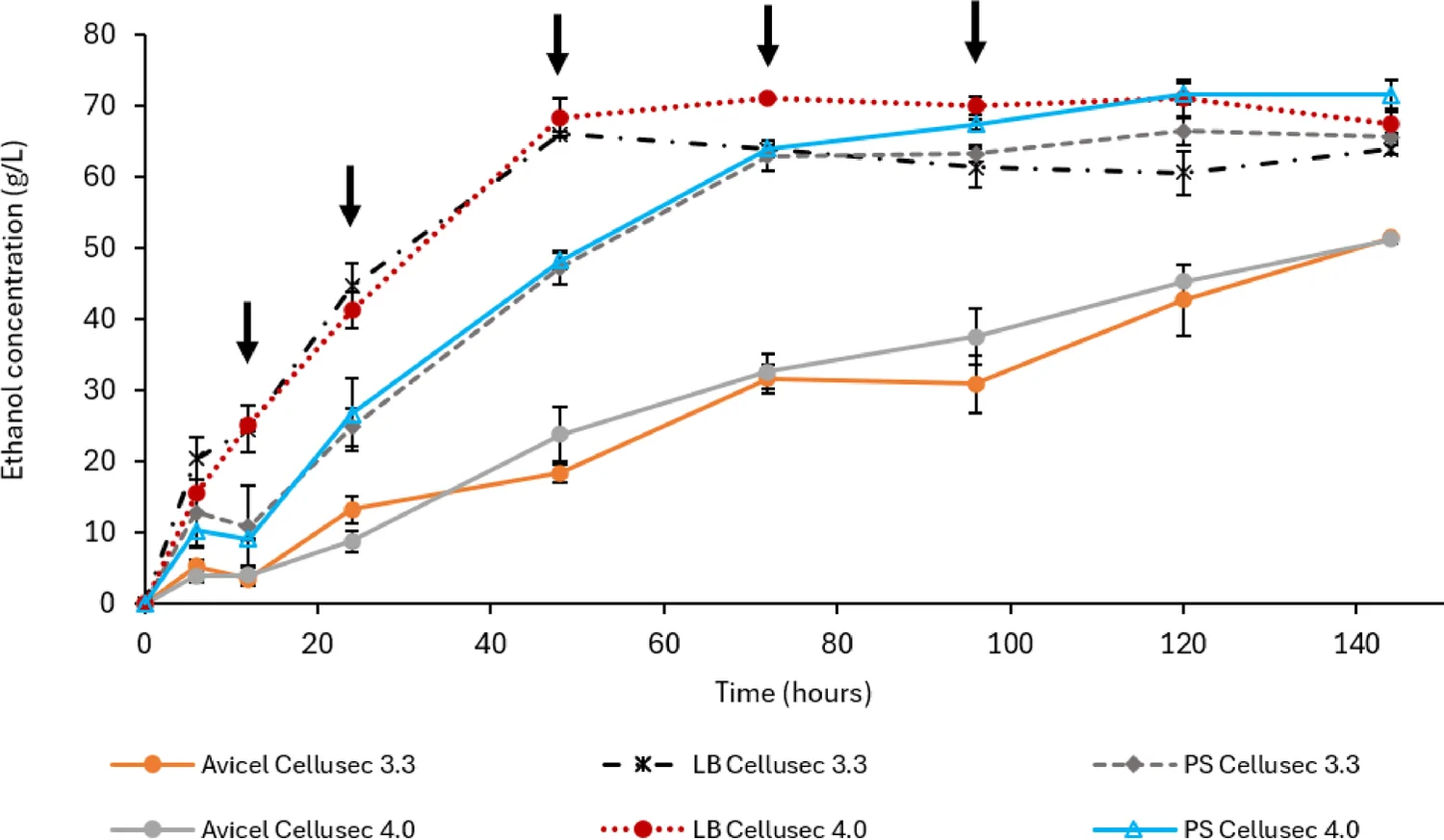
Ethanol production by Cellusec^®^ 3.3 (Avicel^®^ cellulose indicated by filled triangles, LB indicated by x, PS indicated by diamonds), Cellusec^®^ 4.0 (Avicel^®^ cellulose indicated by circles, LB indicated by circles and dotted line, PS indicated by unfilled triangles) using Avicel^®^ cellulose compared to using LB and PS during high-solids fed-batch SScF at 37 °C, at a 30% (w/w) final dry solid loading and a Cellic^®^ CTec3-HS dosage of 5 FPU/g ds. Error bars represent the standard deviation of triplicate experiments. Arrows represent when 5% (w/w) dry solids were fed

The xylose fermenting capabilities of the advanced CBP yeast strains, Cellusec^®^ provided substantial improvement to the ethanol production (Fig. 1). Evidently, there was significant (*p* < 0.05) improvement in the ethanol titres when Cellusec^®^ 3.3 and 4.0 were used to ferment LB and PS compared to Avicel^®^ cellulose.

In general, the ethanol production by Cellusec^®^ 4.0 was more stable compared to Cellusec^®^ 3.3. This was observed when the ethanol concentration from fermenting LB using Cellusec^®^ 3.3 was significantly (*p* < 0.05) lower than using Cellusec^®^ 4.0 from 72 to 120 h (Fig. 1). Similarly, the ethanol production of Cellusec^®^ 4.0 was marginally higher than that of Cellusec^®^ 3.3 when Avicel^®^ cellulose and PS were fermented. This indicated that certain shortcomings of Cellusec^®^ 3.3 were improved in Cellusec^®^ 4.0.

### Fermentation profiles comparing the effect of media on fermentation performance of *S. cerevisiae* Cellusec^®^ 2.0, 3.3 and 4.0 in vertical fermentation vessels

**Fig. 2:**
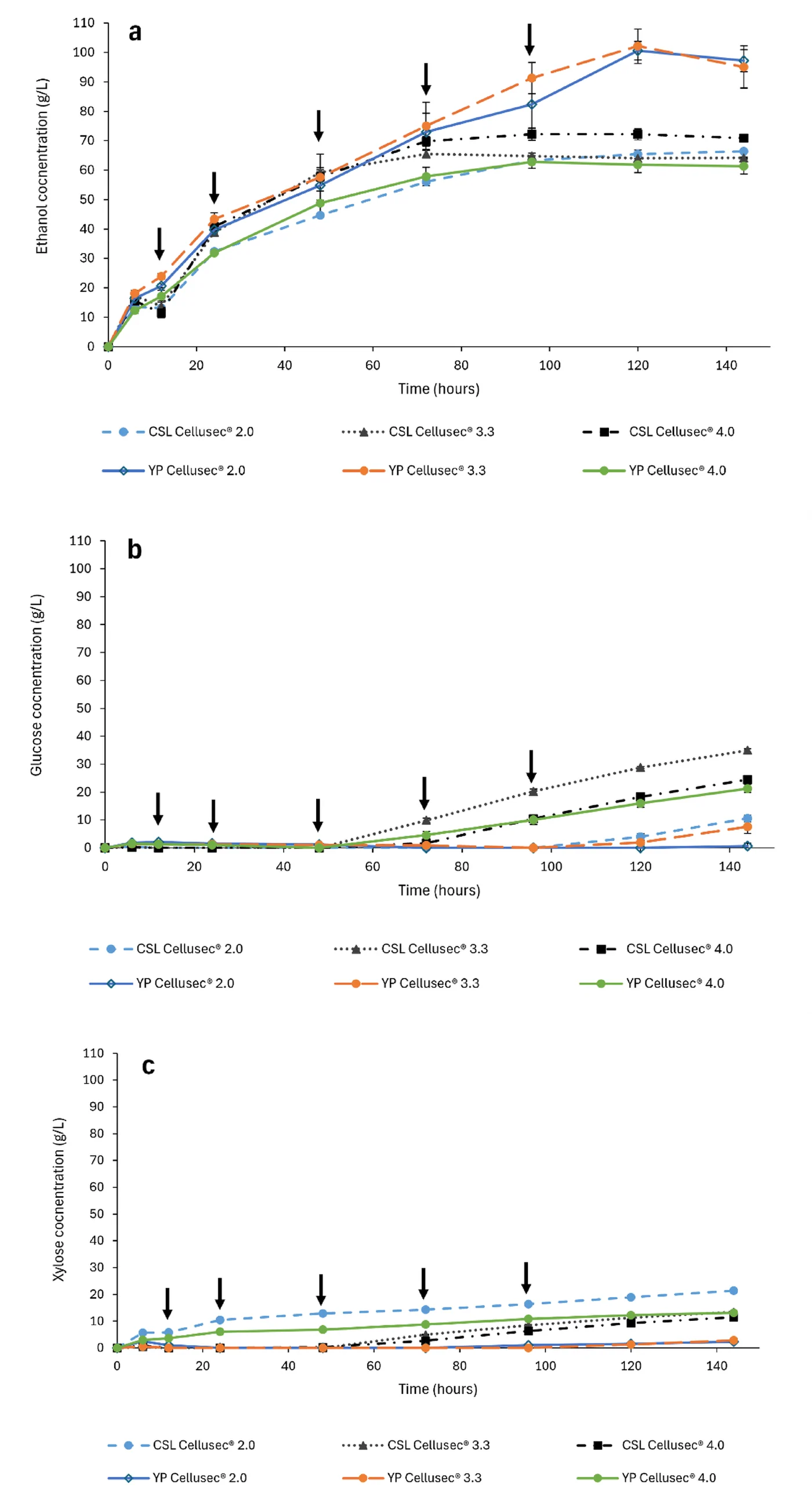
Ethanol (**a**) concentration evolution profile and glucose (**b**) and xylose (**c**) concentrations (g/L) observed during fermentation of LB at 37 °C, using Cellusec^®^ 2.0 (CSL indicated by circles, YP indicated by diamond), Cellusec^®^ 3.3 (CSL indicated by triangles, YP indicated by circles) and Cellusec^®^ 4.0 (CSL indicated by squares, YP indicated by circles) in CSL and YP media in high-solids fed-batch SScF, at a 30% (w/w) final dry solid loading and Cellic^®^ CTec3-HS dosage of 3.5 FPU/g ds. Error bars represent the standard deviation of triplicate experiments. Arrows represent when 5% (w/w) dry solids were fed

Using a less nutrient dense medium, such as the CSL-based medium compared to a more nutrient dense medium, such as YP medium may reduce the cost of waste paper-to-ethanol [54]. The ethanol concentrations for Cellusec^®^ 2.0 and 3.3 were higher when the YP medium was used instead of the CSL medium, while for Cellusec^®^ 4.0 a lower ethanol concentration was observed (Fig. 2a). Moreover, Cellusec^®^ 4.0 performed better than Cellusec^®^ 2.0 and 3.3 when the more cost effective, less nutrient dense medium CSL-based medium was used, which may indicate a reduced dependency on a more nutrient dense medium source such as YP medium.

Glucose accumulation (Fig. 2b) for Cellusec^®^ 2.0, 3.3 and 4.0 was noted with the CSL and YP media, indicating a higher rate of glucose production compared to the rate at which the yeast consumed it. The glucose accumulation from Cellusec^®^ 3.3 was the highest when compared to Cellusec^®^ 2.0 and 4.0 using the CSL medium. Similarly, Bunga et al., [48] reported residual glucose concentrations of 45 g/L present during fermentation using Cellusec^®^ 3.3. The CBP yeast strains Cellusec^®^ 3.3 and Cellusec^®^ 4.0 displayed a similar trend in glucose and xylose accumulation starting at 48 h, which was expected as Cellusec^®^ 4.0 was derived from Cellusec^®^ 3.3.

The xylose accumulation (Fig. 2c) using Cellusec^®^ 2.0 and 3.3 was minimal when the YP medium was used. While the opposite was noted for Cellusec^®^ 4.0, which had a higher xylose accumulation when the YP medium was used compared to the CSL medium. Evident from Fig. 2, using Cellusec^®^ 2.0 and 3.3 with a CSL-based medium and Cellusec^®^ 4.0 with a YP medium had a greater inhibitory effect as the ethanol production stopped while the sugar accumulation occurred. This could suggest that yeast prioritised adaptation and survival above carbon and protein metabolism [64].

### Lab-scale high-solids fed-batch SScF in a vertical fermentation vessel

**Fig. 3:**
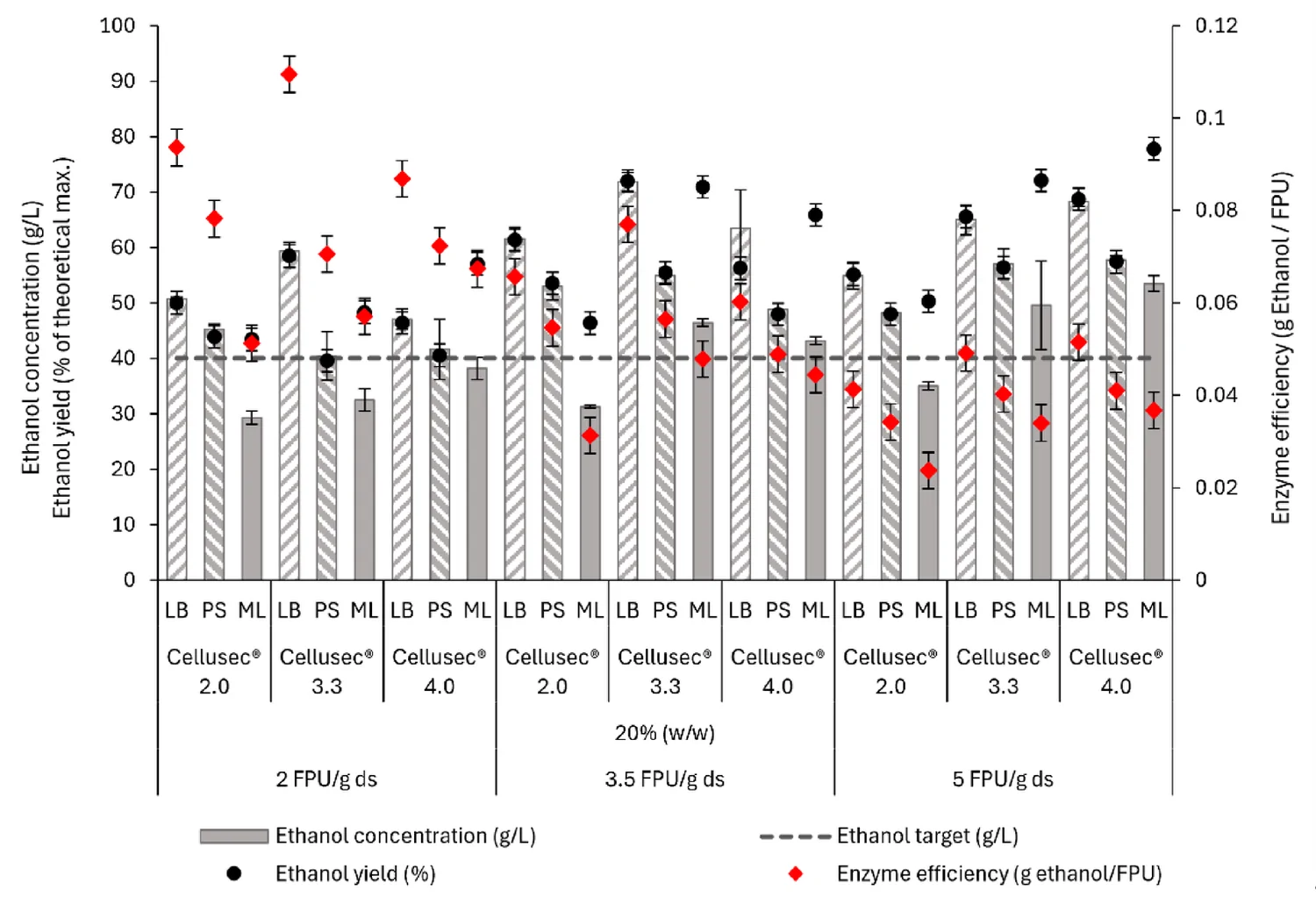
Maximum ethanol concentration (g/L) (indicated by bars), ethanol yield (% of theoretical max.) (indicated by circles) and enzyme efficiency (g ethanol/FPU) (indicated by diamonds) of *S. cerevisiae* strains Cellusec^®^ 2.0, 3.3, and 4.0 after 120 h fermentation, using LB, PS and ML in high-solids fed-batch SScF at 20% (w/w) final dry solid loading and Cellic^®^ CTec3-HS dosages of 2, 3.5 and 5 FPU/g ds. The broken line represents the target ethanol concentration of 40 g/L. Error bars represent the standard deviation of triplicate experiments

**Fig. 4:**
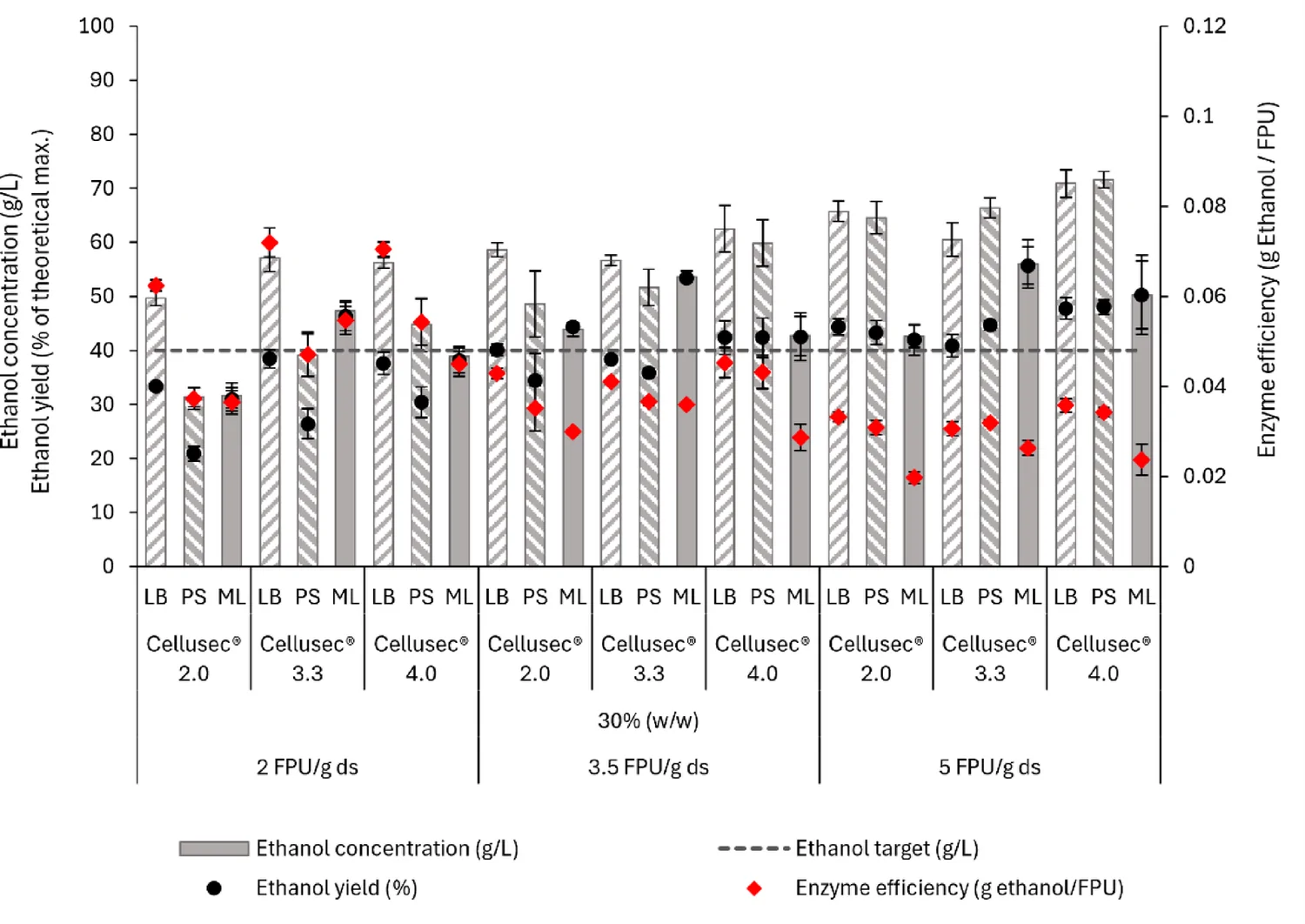
Maximum ethanol concentration (g/L) (indicated by bars), ethanol yield (% of theoretical max.) (indicated by circles) and enzyme efficiency (g ethanol/FPU) (indicated by diamonds) of *S. cerevisiae* strains Cellusec^®^ 2.0, 3.3 and 4.0 after 120 h fermentation, using LB, PS and ML in high-solids fed-batch SScF at 30% (w/w) final dry solid loading and Cellic^®^ CTec3-HS dosages of 2, 3.5 and 5 FPU/g ds. The broken line represents the target ethanol concentration of 40 g/L. Error bars represent the standard deviation of triplicate experiments

High-solids fed-batch SScF in shake flasks were conducted at 20% (w/w) and 30% (w/w) final dry solid loadings indicated in Figs. 3 and 4, respectively. A comparison was conducted between the fermentation performance of advanced *S. cerevisiae* CBP yeast strains Cellusec^®^ 2.0, 3.3 and 4.0 on fermenting LB, PS and ML while simultaneously investigating reduced exogenous enzyme dosages (2, 3.5 and 5 FPU/g ds). Furthermore, Table 1 displays the fermentation performance of fed-batch SScF in shake flasks at 20% (w/w) and 30% (w/w) final dry solid loadings at an enzyme dosage of 2 FPU/g ds for a direct comparison.

#### High-solids fed-batch SScF at 20% (w/w) solid loading

Generally, LB outperformed the other waste paper streams in terms of ethanol titres, yields, enzyme efficiencies and volumetric productivities for vertical, high-solids fed-batch SScF, regardless of the yeast strain or enzyme dosage (Figs. 3 and 4; Table 1). Specifically, the fermentation of LB at 20% (w/w) solid loading (Fig. 3) resulted in the highest ethanol titre of 71.8 g/L, yield of 72.0% of the theoretical maximum and volumetric productivity of 0.60 g/L/h at an enzyme dosage of 3.5 FPU/g ds using CBP strain Cellusec^®^ 3.3. Whereas at an increased enzyme dosage of 5 FPU/g ds the highest ethanol titres, yields and volumetric productivity were below 57.8 g/L, 77.8% of the theoretical maximum and 0.48 g/L/h, respectively, observed for PS and ML using Cellusec^®^ 4.0 (Fig. 3). The superior performance of LB as a feedstock may be attributed to the higher carbohydrate content of LB when compared to ML, as well as the increased digestibility of LB relative to the PS substrate [35]. On the other hand, although ML had the lowest ethanol titres and enzyme efficiencies compared to LB and PS, the ethanol yields were equivalent or higher than that of LB and PS, indicating a high degree of digestibility for ML. The lower ethanol titres and enzyme efficiencies of ML was likely due to ML being limited by the available quantity of cellulose (30.1% (w/w)) and hemicellulose (11.2% (w/w)) compared to LB and PS with maximum cellulose contents of 51.2% (w/w) and hemicellulose contents of 14.4% (w/w) [35].

The enzyme efficiencies for fed-batch SScF at 20% (w/w) (Fig. 3) and 30% (w/w) (Fig. 4) solid loadings peaked at the lowest enzyme dosage of 2 FPU/g ds, which indicated that more ethanol was produced per FPU of exogenous enzyme added. This implied that operating at 2 FPU/g ds has the potential to improve the cost contribution from enzymes (Fig. S2) [8, 51, 52], despite the up to 1.7-fold greater ethanol titres and yields at the higher enzyme dosages of 3.5 and 5 FPU/g ds. Furthermore, Humbird et al., [53] noted that the enzyme dosage had a nearly linear contribution to the minimum ethanol selling price (MESP) from lignocellulosic biomass, therefore, it is pertinent to use the lowest possible enzyme dosage. At the lowest enzyme dosage of 2 FPU/g ds, LB and PS cultures achieved the minimum-acceptable ethanol titre of 40 g/L, which is required for an industrially viable fermentation process [36–39]. On the other hand, this target was not reached using ML as the substrate (Table 1) at 20% (w/w) or 30% (w/w) solid loadings, except with Cellusec^®^ 3.3.

#### High-solids fed-batch SScF at 30% (w/w) solid loading

Comparing Figs. 3 and 4, generally, an increase from 20% (w/w) to 30% (w/w) solid loading resulted in marginally higher ethanol titres, predominantly at increased enzyme dosages of 3.5 and 5 FPU/g ds (Fig. 4). This was expected as the cultures were provided with a higher quantity of carbohydrates and enzymes. Contrastingly, when evaluating the individual waste paper streams, an increase in solid loadings resulted in a marginal decrease in ethanol titres and volumetric productivity using LB and PS, whereas the converse was apparent using ML (Figs. 3 and 4 and Table 1). This could be attributed to the higher degree of digestibility of ML compared to LB and PS [35], demonstrated by the ethanol yields attained using ML that were equivalent or higher than that of LB and PS (Figs. 3 and 4; Table 1). For LB and PS this was notably evident from the marked decrease in enzyme efficiency, especially at 2 FPU/g ds, where the enzyme efficiency of 0.087–0.109 g ethanol/FPU at 20% (w/w) decreased to 0.062–0.072 g ethanol/FPU at 30% (w/w) for LB, and the enzyme efficiency of 0.071–0.078 g ethanol/FPU at 20% (w/w) decreased to 0.037–0.054 g ethanol/FPU at 30% (w/w) solid loading for PS (Table 1).

Ultimately, since the fermentation performance in terms of ethanol titre, yield and volumetric productivity for high-solids fed-batch SScF of LB, ML and PS were observed to be substrate specific, each feedstock demands different fermentation conditions in terms of solid loading and enzyme dosage. Furthermore, the selection of a preferred enzyme dosage and solid loading for high-solids fed-batch SScF required a compromise between the enzyme efficiency, ethanol concentration and yield for each substrate, also based on the performances of CBP yeasts. The ML waste paper stream performed better at a higher solid loading of 30% (w/w) as it required a higher quantity of carbohydrates to meet the target ethanol concentration of 40 g/L (Fig. 4). Moreover, ML fermented using Cellusec^®^ 3.3 at 30% (w/w) solid loading, indicated no significant (*p* > 0.05) difference in the ethanol titres and yields between 2 and 5 FPU/g ds, thus the lowest enzyme dosage with the highest enzyme efficiency would be preferred. The LB and PS waste paper streams especially, displayed improved ethanol concentrations, volumetric productivities and enzyme efficiencies at 2 FPU/g ds and 20% (w/w) solid loading (Fig. 3; Table 1). This could be due to improved mass transfer (mixing), especially when hydrolysis was significantly limited by the lowest enzyme dosage of 2 FPU/g ds [18, 45].

A comparison of the fermentation performance, specifically at the lowest enzyme dosage (2 FPU/g ds) for the fed-batch SScF process at 20% and 30% (w/w) solid loadings is shown in Table 1. Using Cellusec^®^ 4.0 at the increased solid loading of 30% (w/w) improved the ethanol concentrations and volumetric productivities when compared to 20% (w/w), although these trends were statistically significant (*p* < 0.05) only for LB (Table 1). Improved fermentation performance by Cellusec^®^ 4.0 at an increased solid loading and the lowest enzyme dosage of 2 FPU/g ds, could suggest superior enzyme-production by Cellusec^®^ 4.0 compared to the Cellusec^®^ 2.0 and 3.3 CBP strains also seen in Fig. 1.

It was observed in Figs. 3 and 4; Table 1 that the fermentation of untreated LB, PS and ML substrates using advanced CBP yeast strains displayed better fermentation performance at lower exogenous enzyme dosages (2, 3.5 and 5 FPU/g ds) and solid loadings of 20% and 30% (w/w) than that obtained in the studies by Nishimura et al., [21] and Dey et al., [47] that also used a fed-batch approach (Table 1). This highlighted the benefit of using newer enzyme cocktails with an advanced CBP yeast strain as applied in the current study. Especially, the 32-to-83-fold lower enzyme dosage of 1.8 to 4.7 FPU/g total solids (2 to 5 FPU/g ds) used in the current study compared to 158 FPU/g total solids used in the study by Dey et al., [47].

**Table 1:** Comparison of the fermentation performance of *S. cerevisiae* strains Cellusec^®^ 2.0, 3.3 and 4.0 after 120 h in fed-batch SScF culture in vertical fermentation vessels at 20% (w/w) and 30% (w/w) dry final solid loadings at a Cellic^®^ CTec3-HS dosage of 2 FPU/g ds with LB, PS and ML waste-paper substrates (Standard deviations are presented in brackets)

Substrate	Strain	Process configuration	Enzyme dosage (enzyme)	Solid loading (% w/w)	Max. ethanol titre (g/L)	Enzyme efficiency (g ethanol/FPU)	Ethanol yield (% of theoretical max.)	Volumetric productivity (g ethanol /L h)	Ethanol yield (g ethanol/g carbohydrates)	Refere nces
LB	Cellusec^®^ 2.0	Fed-batch SScF	(Cellic CTec3-HS) LB: 2 FPU/g ds 1.8 FPU/ g total solids^a^ 3.1 FPU/g carbohydrates^a^ PS: 2 FPU/g ds 1.8 FPU/ g total solids^a^ 3.2 FPU/g carbohydrates^a^ ML: 2 FPU/g ds 1.9 FPU/ g total solids^a^ 4.8 FPU/g carbohydrates^a^	20	50.7 (0.4)	0.094 (0.001)	50.0 (0.4)	0.42 (0.003)	0.29 (0.002)	This study
				30	49.7 (1.3)	0.062 (0.001)	33.3 (0.7)	0.41 (0.01)	0.17 (0.01)	
	Cellusec^®^ 3.3			20	59.3 (1.6)	0.109 (0.003)	58.5 (1.8)	0.49 (0.01)	0.33 (0.01)	
				30	57.2 (2.6)	0.072 (0.003)	38.4 (1.7)	0.48 (0.02)	0.21 (0.003)	
	Cellusec^®^ 4.0			20	47.1 (1.8)	0.087 (0.003)	46.4 (1.5)	0.39 (0.02)	0.27 (0.01)	
				30	56.2 (1.0)	0.070 (0.002)	37.6 (2.1)	0.47 (0.01)	0.21 (0.003)	
PS	Cellusec^®^ 2.0			20	45.2 (1.0)	0.078 (0.003)	43.9 (1.4)	0.38 (0.01)	0.25 (0.01)	
				30	31.3 (1.8)	0.037 (0.002)	20.9 (1.3)	0.26 (0.02)	0.14 (0.02)	
	Cellusec^®^ 3.3			20	40.4 (4.4)	0.071 (0.008)	39.6 (4.5)	0.34 (0.04)	0.23 (0.03)	
				30	39.2 (3.9)	0.047 (0.005)	26.4 (2.8)	0.33 (0.03)	0.15 (0.001)	
	Cellusec^®^ 4.0			20	41.4 (5.7)	0.072 (0.01)	40.3 (5.6)	0.34 (0.05)	0.23 (0.03)	
				30	44.7 (2.9)	0.054 (0.005)	30.4 (2.9)	0.37 (0.04)	0.16 (0.004)	
ML	Cellusec^®^ 2.0			20	29.3 (1.2)	0.051 (0.002)	43.4 (1.7)	0.24 (0.01)	0.25 (0.01)	
				30	31.6 (2.3)	0.037 (0.003)	30.9 (2.2)	0.26 (0.02)	0.23 (0.01)	
	Cellusec^®^ 3.3			20	32.5 (2.0)	0.057 (0.003)	48.3 (2.8)	0.27 (0.02)	0.28 (0.02)	
				30	47.3 (1.8)	0.055 (0.003)	46.3 (2.6)	0.39 (0.02)	0.30 (0.007)	
	Cellusec^®^ 4.0			20	38.2 (2.0)	0.067 (0.004)	57.1 (3.1)	0.32 (0.02)	0.33 (0.02)	
				30	38.9 (1.9)	0.045 (0.003)	38.1 (2.3)	0.32 (0.02)	0.27 (0.01)	
Pretreated copy paper	*S. cerevisiae* KF-7 (derived from flocculating & thermotolerant yeast strains)	PSSF: batch	40 FPU/g ds (Cellic CTec2)	15	32	-	91.8	0.53		[21]
		PSSF: Fed-batch	13 FPU/g ds (Cellic CTec2)	55	41.8	-	83.8	0.50		
Primary sludge from paper and pulp mills	*S. cerevisiae* ATCC^®^26602 (xylose not fermented)	SSF: batch	15 FPU/g carbohydrates (cellulolytic NS 22192 & xylanolytic Cellic^®^ HTec2,)	22	59.1	-	86.3	1.97		[13]
Pulp and paper sludge	*Pichia stipitis* NCIM-3499 & *S. cerevisiae* (glucose & xylose fermented)	PSSF: fed-batch during pre-saccharification	158 FPU/g total solids (Crude cellulase enzyme, SUPERCUT)	18	42.34	-	0.53 g ethanol/g glucose and xylose^b^	0.71 (only fermentation) 0.39 (pre-saccharification + fermentation)		[47]

The data from the present study is compared to the fermentation performance of paper-based substrates reported in literature

^a^ Variations in enzyme dosage between LB, ML and PS are due to differences in moisture content and carbohydrate content

^b^The basis of the ethanol yield from this study differs from the current study

Lastly, from a waste valorisation point of view, it is preferred to convert as much of the waste stream as possible. Therefore, fed-batch SScF at 20% (w/w) and 2 FPU/g ds is more attractive than at 30% (w/w) and 2 FPU/g ds. This was indicated by the lower ethanol yields observed at 30% (w/w), highlighting a reduced cellulose and hemicellulose conversion to ethanol (Table 1), resulting in a substantial amount of unutilised substrate. Although, operating at a higher solid loading allows for a larger quantity of substrate to be processed per unit reactor volume and reduces the required reactor size and process water which in turn reduces the capital and operating costs [42, 50, 65]. A low conversion of substrate to product, illustrated by product yield (as g ethanol per g carbohydrate in material) (Table 1) indicates inefficient utilisation of the substrate. Furthermore, in the study by Wang et al., [66], it was reported that the cost benefits of a higher ethanol yield outweighed the additional capital cost incurred for increased reactor capacity when operating at a longer residence time. Thus, considering the need to simultaneously maximise the enzyme efficiency, ethanol yield and volumetric productivity, while ensuring that the minimum-required ethanol concentration of 40 g/L is achieved [52, 67, 68] the fed-batch SScF at 20% (w/w) dry final solid loading would be the preferred option.

### Lab-scale high-solids batch SScF in a horizontal fermentation vessel

**Fig. 5:**
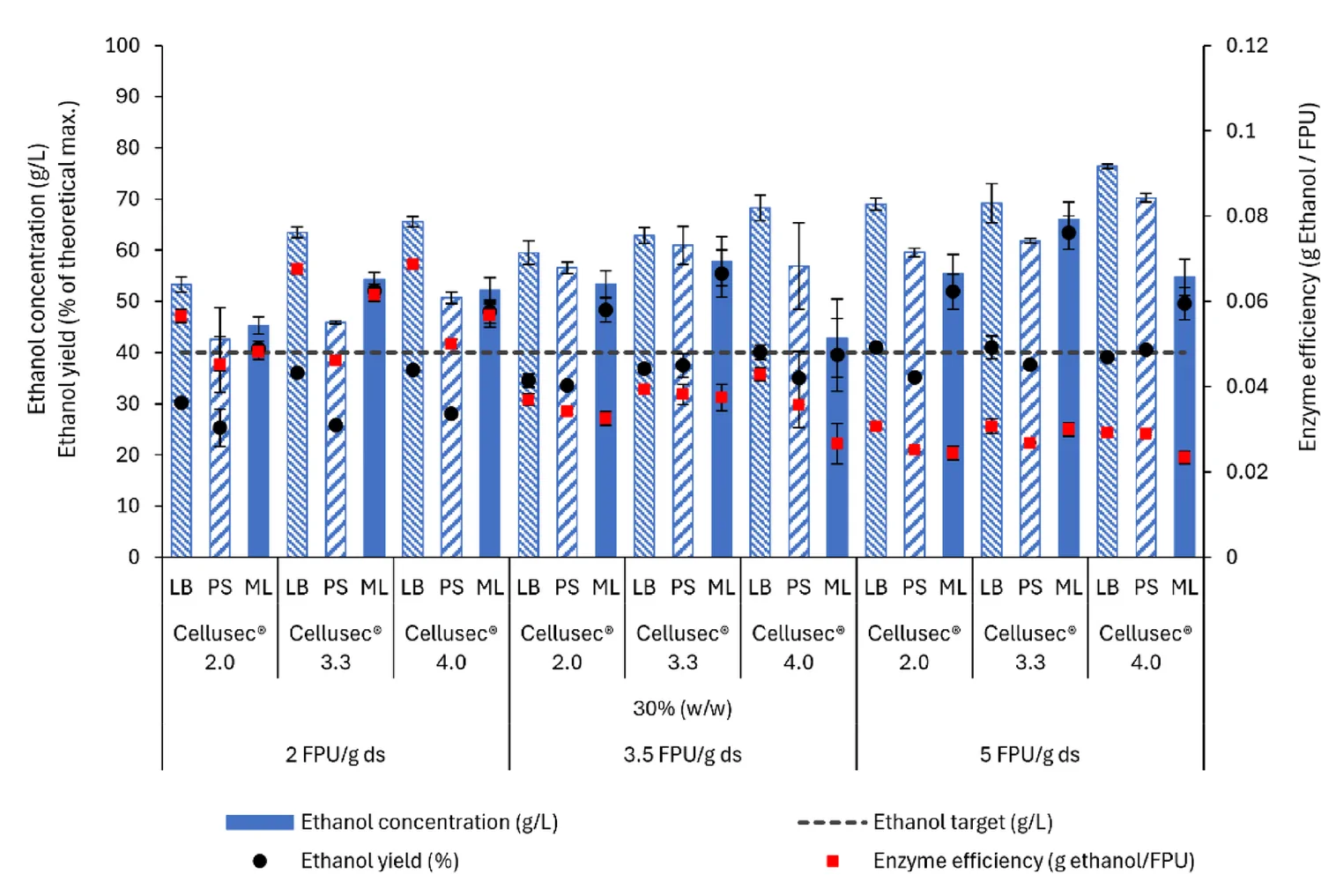
Maximum ethanol concentration (g/L) (indicated by bars), ethanol yield (% of theoretical max.) (indicated by circles) and enzyme efficiency (g ethanol/FPU) (indicated by squares) of *S. cerevisiae* strains Cellusec^®^ 2.0, 3.3 and 4.0 after 120 h fermentation, using LB, PS and ML in high-solids batch SScF at 30% (w/w) final dry solid loading and Cellic^®^ CTec3-HS dosages of 2, 3.5 and 5 FPU/g ds. The broken line represents the target ethanol concentration of 40 g/L. Error bars represent the standard deviation of triplicate experiments

High-solids batch SScF in roller bottles was conducted at 30% (w/w) and 40% (w/w) dry solid loadings, indicated in Figs. 5 and 7, respectively. A comparison was conducted between the fermentation performance of advanced *S. cerevisiae* CBP yeast strains Cellusec^®^ 2.0, 3.3 and 4.0 when fermenting LB, PS and ML and simultaneously investigating reduced exogenous enzyme dosages of 2, 3.5 and 5 FPU/g ds. "Sugar accumulation profiles in high-solids batch SScF in horizontal fermentation vessels" indicates the sugar accumulation accrued in high-solids batch SScF. Furthermore, Table 2 displays the fermentation performance of batch SScF in horizontal roller bottles at 30% (w/w) and 40% (w/w) dry solid loadings at an enzyme dosage of 2 FPU/g ds for a direct comparison.

#### High-solids batch SScF at 30% (w/w) solid loading

Within the selected range of batch SScF conditions shown in Figs. 5 and 7, using a horizontal bioreactor at solid loadings of 30% and 40% (w/w) resulted in high ethanol titres of 42.6–80.4 g/L, exceeding the threshold ethanol concentration. Furthermore, the ethanol titres obtained using high-solids batch SScF in the roller bottles (Fig. 5) were more consistent between the different waste paper substrates than the high-solids fed-batch SScF using shake flasks (Fig. 4).

Similar to the high-solids fed-batch SScF discussed in "Lab-scale high-solids fed-batch SScF in a vertical fermentation vessel", the highest enzyme efficiencies for the high-solids batch SScF were calculated at the lowest enzyme dosage of 2 FPU/g ds (Figs. 5 and 7). This correspondingly indicated that operating at a reduced exogenous enzyme dosage of 2 FPU/g ds may improve the cost of the fermentation process by reducing the cost contribution from exogenous enzyme addition (Fig. S4). This was evident in Figs. 5 and 7, where an increased enzyme dosage resulted in a decreased enzyme efficiency. For example, at 30% (w/w) solid loading shown in Fig. 5, LB fermented with Cellusec^®^ 3.3 resulted in a 52.6% decrease in the enzyme efficiency, from 0.068 to 0.039 g ethanol/FPU when the enzyme dosage was increased from 2 to 3.5 FPU/g ds. This was in line with the findings by Huang et al., [69] and van Dyk et al., [12].

In the current study, the solid loading of 30% (w/w) was higher while the enzyme dosages of 2 to 5 FPU/g ds (3 to 12 FPU/g carbohydrates) were 1.2-to-5-fold lower compared to batch SSF of 22% (w/w) solids and 15 FPU/g carbohydrates (Table 1) in the study by Mendes et al., [13]. Additionally, the benefit of using an advanced CBP *S. cerevisiae* strain at 30% (w/w) solid loading and newer enzyme cocktail (Cellic^®^ CTec3-HS) at 2 FPU/g ds (a 20-fold lower enzyme dosage compared to 40 FPU/g ds [21]) was highlighted. This was indicated by the ethanol titres obtained in the current study exceeding the ethanol titres obtained in the study by Nishimura et al., [21] using batch PSSF with 15% (w/w) solid loading and an enzyme dosage of 40 FPU/g ds (Table 1).

#### Sugar accumulation profiles in high-solids batch SScF in horizontal fermentation vessels

**Fig. 6:**
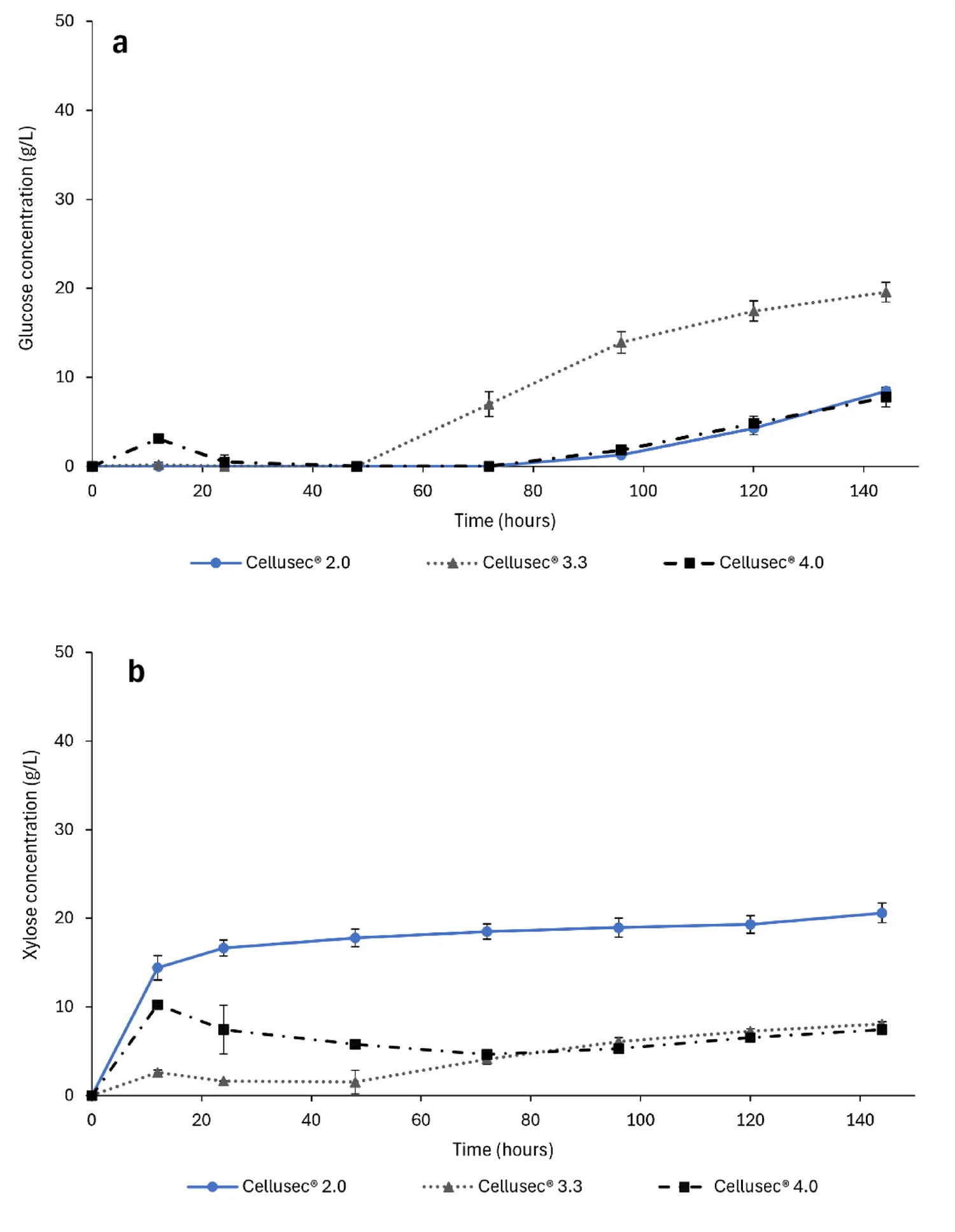
Glucose (**a**) and xylose (**b**) concentrations (g/L) observed during fermentation using *S. cerevisiae* strains Cellusec^®^ 2.0 (indicated by circles), Cellusec^®^ 3.3 (indicated by triangles) and Cellusec^®^ 4.0 (indicated by squares) after 144 h, using LB at 37 °C in high-solids batch SScF at 30% (w/w) dry solids loading and Cellic^®^ CTec3-HS dosage of 3.5 FPU/g ds. Error bars represent the standard deviation of triplicate experiments

The sugar accumulation in high-solids batch SScF (horizontal bioreactor) (Fig. 6) was lower than the sugar accumulation observed in high-solids fed-batch SScF (vertical bioreactor) shown in Fig. 2b, c. However, regardless of the fermentation configuration, the glucose accumulation using Cellusec^®^ 3.3 started at 48 h. Furthermore, Fig. 6 depicted that the highest glucose accumulation was observed using Cellusec^®^ 3.3, while the highest xylose accumulation resulted from using Cellusec^®^ 2.0 during batch SScF at 30% (w/w) solid loading and 3.5 FPU/g ds. This was similar to the observations from Fig. 2b, c for fed-batch SScF (vertical bioreactor) at the same solid loading and enzyme dosage.

Moreover, fermenting LB using Cellusec^®^ 4.0 resulted in the highest ethanol titre of 68.3 g/L (Fig. 5) compared to Cellusec^®^ 2.0 (59.5 g/L) and Cellusec^®^ 3.3 (62.8 g/L) at 30% (w/w) solid loading and 3.5 FPU/g ds, with lower glucose accumulation compared to Cellusec^®^ 3.3 and lower xylose accumulation compared to Cellusec^®^ 2.0 (Fig. 6). These observations further demonstrated that Cellusec^®^ 4.0 was the preferred CBP yeast strain.

#### High-solids batch SScF at 40% (w/w) solid loading

**Fig. 7:**
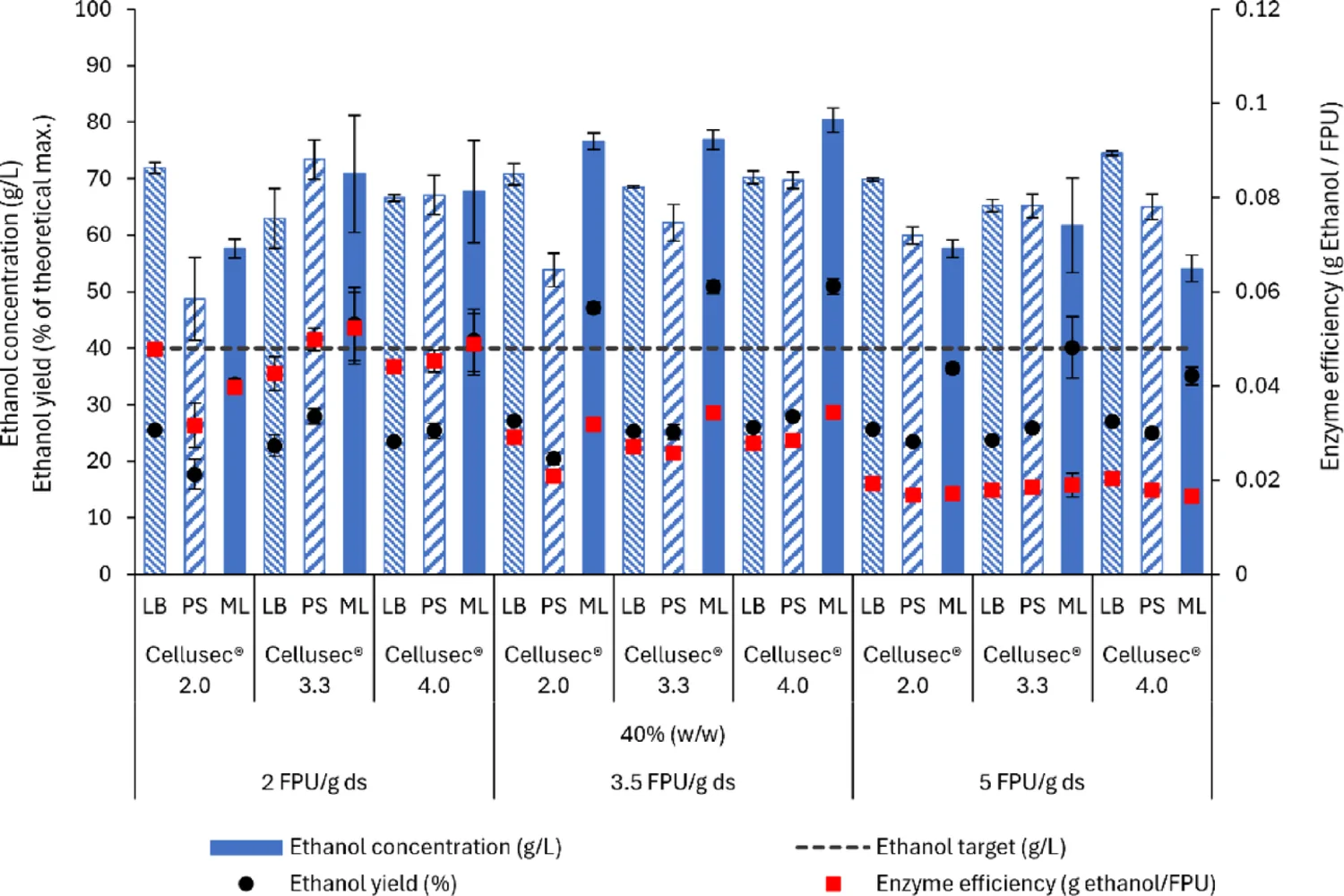
Maximum ethanol concentration (g/L) (indicated by bars), ethanol yield (% of theoretical max.) (indicated by circles) and enzyme efficiency (g ethanol/FPU) (indicated by squares) of *S. cerevisiae* strains Cellusec^®^ 2.0, 3.3 and 4.0 after 120 h fermentation, using LB, PS and ML in high-solids batch SScF at 40% (w/w) final dry solid loading and Cellic^®^ CTec3-HS dosages of 2, 3.5 and 5 FPU/g ds. The broken line represents the target ethanol concentration of 40 g/L. Error bars represent the standard deviation of triplicate experiments

Increasing the solid loading from 30% to 40% (w/w) in the high-solids batch SScF process (Fig. 7) resulted in improved ethanol titres, with proportionally lower ethanol yields and enzyme efficiencies. As shown in Fig. 7, the ethanol titres were high (between 48.7 and 80.4 g/L), while the ethanol yields (17.7% – 50.9% of the theoretical maximum) and enzyme efficiencies (0.016–0.052 g ethanol/FPU) were low, compared to 30% (w/w) solid loading (Fig. 5) that had ethanol titres between 42.6 and 76.4 g/L, ethanol yields between 25.3% − 63.5% of the theoretical maximum and enzyme efficiencies between 0.023 and 0.069 g ethanol/FPU. This indicated poor mass transfer at an increased solid loading of 40% (w/w). As shown in Figs. 5 and 7, an impediment of the high-solids batch SScF process in the horizontal bioreactor was the low ethanol yields. Nonetheless, it was quite impressive that at low enzyme dosages of 2 to 5 FPU/g dsand high solid loadings of 30–40% (w/w), high ethanol titres up to 80.4 g/L were achieved using a batch SScF approach, which in part may be attributed to the free fall mixing motion in the horizontal bioreactor [43].

At high solids loadings of 30% and 40% (w/w) (Figs. 5 and 7), increasing the enzyme dosage, within the low range of 2–5 FPU/g ds, generally resulted in marginally improved ethanol titres and yields. However, the inverse was observed for ML, specifically, at 40% (w/w) solid loading when the enzyme dosage was increased from 3.5 to 5 FPU/g ds (Fig. 7). In fact, the fermentation performance was greater at 2 FPU/g ds compared to 5 FPU/g ds, which also had a large effect on the enzyme cost (Fig. S5). For example, when fermenting ML with Cellusec^®^ 4.0 at 2, 3.5 and 5 FPU/g ds, the ethanol concentrations were 67.7, 80.4 and 54.1 g/L, respectively, with a 20.1% decrease in ethanol concentration when the enzyme dosage was increased from 2 to 5 FPU/g ds, and a 32.7% decrease when the enzyme dosage was increased from 3.5 to 5 FPU/g ds. The enzyme costs were $0.42, $0.60 and $1.24/L ethanol, respectively, with an increase of 194.9% from 2 to 5 FPU/g ds and a 107.3% increase from 3.5 to 5 FPU/g ds (Fig. S5). A similar trend was observed for LB fermented with Cellusec^®^ 4.0 and PS fermented with Cellusec^®^ 2.0. Furthermore, increasing the enzyme dosage from 2 to 5 FPU/g ds resulted in the enzyme efficiency decreasing from 0.049 to 0.017 g ethanol/FPU, which, despite increased enzyme addition resulted in little to no improvements in the ethanol production. This was also noted in previous studies, that considered the ethanol fermentation of lignocellulosic substrates such as paper sludge [12] and corn cobs [69], where increasing the enzyme dosage did not result in significantly improved ethanol titres. An interesting observation was that ML maintained its high degree of digestibility, observed from the higher ethanol yields compared to LB and PS (Fig. 7). The decrease in ethanol concentrations at increased enzyme dosages could likely be that improved enzyme hydrolysis at 5 FPU/g ds combined with poor mixing at 40% (w/w) solid loading, resulted in inhibition of enzyme activity at high sugar concentrations [70–72]. Another possible reason could be that at the higher enzyme dosage of 5 FPU/g ds, improved enzymatic hydrolysis could possibly result in a larger quantity of paper fillers (clay and kaolin) being released. Literature stated that paper fillers have been noted to preferentially adsorb enzymes, impeding the efficiency of hydrolysis [73–76].

The preferred solid loading for high-solids batch SScF in a horizontal vessel was determined to be substrate specific. A general observation (Figs. 5 and 7) was that the ethanol titres and volumetric productivities, were generally higher at 40% (w/w), while the enzyme efficiencies and ethanol yields were higher at 30% (w/w). A decrease in the ethanol yields as the solid loading was increased was also noted in literature [17, 45, 50]. When fermenting LB and PS, a solid loading of 30% (w/w) was preferred since there was no significant difference (*p* > 0.05) in the ethanol concentrations and volumetric productivities between 30% and 40% (w/w) solid loading and the enzyme efficiencies and ethanol yields were generally significantly (*p* < 0.05) higher at 30% compared to 40% (w/w) solid loading. Contrastingly, ML generally had significantly higher ethanol concentrations and volumetric productivities at 40% (w/w) solid loading (Fig. 7), and since the enzyme efficiencies and ethanol yields were not significantly (*p* > 0.05) higher at 30% (w/w), operating at 40% (w/w) solid loading would be preferred for ML.

### Selection of preferred high-solids hydrolysis-fermentation process

It should be noted that the high-solids fed-batch SScF approach in the vertical reactor and the high-solids batch SScF approach in the horizontal reactor are not directly compared, since these systems differ in mode (batch and fed-batch), reactor geometry (vertical and horizontal) and solid loading. Instead, the aim was to identify the better performing system for valorisation of the studied paper waste streams.

**Fig. 8:**
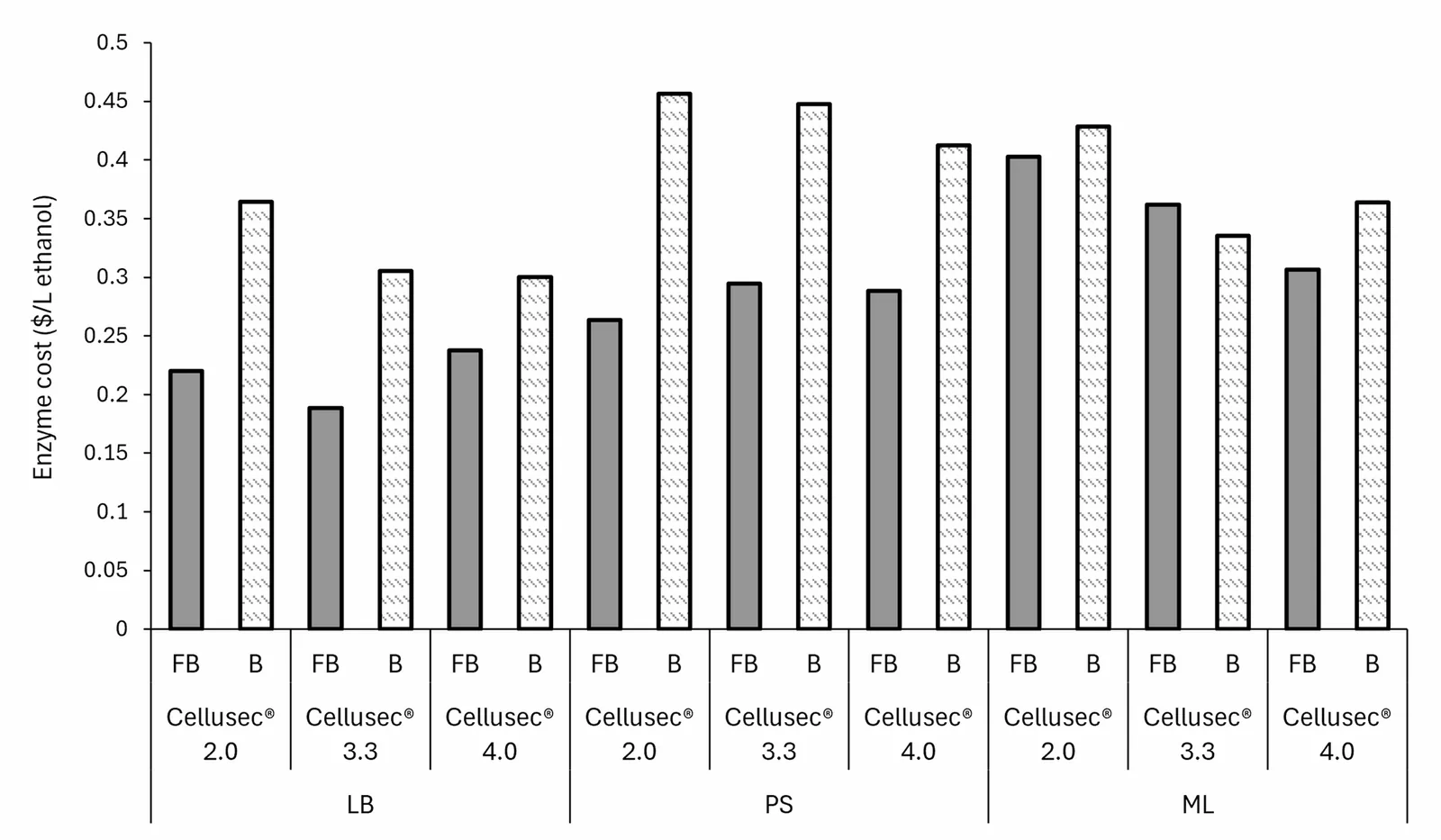
Enzyme cost ($/L ethanol) for fed-batch SScF culture (vertical bioreactor) at 20% (w/w) solid loading (indicated by FB) and batch SScF (horizontal bioreactor) at 30% (w/w) solid loading (indicated by B) at a Cellic^®^ CTec3-HS dosage of 2 FPU/g ds with LB, PS and ML waste paper substrates

The enzyme cost and fermentation parameters: enzyme efficiency, ethanol concentration, ethanol yield and volumetric productivity were analysed for the 20% (w/w) high-solids fed-batch SScF and 30% (w/w) high-solids batch SScF, shown in Table 2 and Fig. 8. These configurations were determined to be the more viable processes for the majority of waste paper substrates for fed-batch and batch SScF, as discussed in the previous "Lab-scale high-solids fed-batch SScF in a vertical fermentation vessel" and "Lab-scale high-solids in batch SScF in a horizontal fermentation vessel", respectively. Preference was given to enzyme efficiency and ethanol yield since they have the greatest effect on the economic viability of a fermentation process [52, 53, 65] while ensuring that the ethanol concentration threshold of 40 g/L was reached [67, 68].

**Table 2:** Fermentation performance of *S. cerevisiae* strains Cellusec^®^ 2.0, 3.3 and 4.0 after 120 h in fed-batch SScF culture (vertical bioreactor) at 20% (w/w) solid loading and batch SScF (horizontal bioreactor) at 30% (w/w) solid loading and a Cellic^®^ CTec3-HS dosage of 2 FPU/g ds with LB, PS and ML waste paper substrates (standard deviations are presented in brackets)

Substrate	Strain		Fermentation mode	Solid loading (% w/w)	Max. ethanol titre (g/L)		Enzyme efficiency (g ethanol/FPU)	Ethanol yield (% of theoretical max.)	Volumetric productivity (g ethanol /L h)
LB	Cellusec^®^ 2.0		Fed-batch	20	50.7 (0.4)		0.094 (0.001)	50.0 (0.4)	0.42 (0.003)
			Batch	30	53.3 (1.5)		0.057 (0.002)	30.2 (0.8)	0.44 (0.01)
	Cellusec^®^ 3.3		Fed-batch	20	59.3 (1.6)		0.109 (0.003)	58.5 (1.8)	0.49 (0.01)
			Batch	30	63.5 (1.1)		0.068 (0.001)	36.1 (0.6)	0.53 (0.01)
	Cellusec^®^ 4.0		Fed-batch	20	47.1 (1.8)		0.087 (0.003)	46.4 (1.5)	0.39 (0.02)
			Batch	30	65.6 (1.1)		0.069 (0.001)	36.7 (0.6)	0.55 (0.01)
PS	Cellusec^®^ 2.0		Fed-batch	20	45.2 (1.0)		0.078 (0.003)	43.9 (1.4)	0.38 (0.01)
			Batch	30	42.6 (6.2)		0.045 (0.007)	25.3 (3.7)	0.35 (0.05)
	Cellusec^®^ 3.3		Fed-batch	20	40.4 (4.4)		0.071 (0.008)	39.6 (4.5)	0.34 (0.04)
			Batch	30	45.9 (0.3)		0.046 (0.0003)	25.8 (0.2)	0.38 (0.002)
	Cellusec^®^ 4.0		Fed-batch	20	41.4 (5.7)		0.072 (0.01)	40.3 (5.6)	0.34 (0.05)
			Batch	30	50.7 (1.1)		0.050 (0.001)	28.0 (0.6)	0.42 (0.01)
ML	Cellusec^®^ 2.0		Fed-batch	20	29.3 (1.2)		0.051 (0.002)	43.4 (1.7)	0.24 (0.01)
			Batch	30	45.3 (1.7)		0.048 (0.002)	40.8 (1.5)	0.38 (0.01)
	Cellusec^®^ 3.3		Fed-batch	20	32.5 (2.0)		0.057 (0.003)	48.3 (2.8)	0.27 (0.02)
			Batch	30	54.3 (1.3)		0.062 (0.002)	52.1 (1.3)	0.45 (0.01)
	Cellusec^®^ 4.0		Fed-batch	20	38.2 (2.0)		0.067 (0.004)	57.1 (3.1)	0.32 (0.02)
			Batch	30	52.2 (2.5)		0.057 (0.003)	48.0 (2.3)	0.44 (0.02)

Overall, the more viable fermentation configuration in terms of the highest enzyme efficiencies and ethanol yields was high-solids fed-batch SScF in the vertical bioreactor at 20% (w/w) solid loading and 2 FPU/g ds. As displayed in Table 2, the ethanol titres obtained during high-solids batch SScF in the horizontal bioreactor at 2 FPU/g ds were generally higher (42.6–63.5 g/L) than those obtained during high-solids fed-batch SScF in the vertical bioreactor (29.3–59.3 g/L). This could be due to the higher quantity of carbohydrates at 30% compared to 20% (w/w) solid loading. However, fed-batch SScF at 20% (w/w) solid loading in the vertical bioreactor achieved higher enzyme efficiencies and ethanol yields (Table 2). This could suggest improved mass transfer in the vertical bioreactor in high-solids fed-batch SScF compared to the high-solids batch SScF in the horizontal bioreactor. It was noted for the LB and PS waste paper streams, that irrespective of the *S. cerevisiae* strain Cellusec^®^ 2.0, 3.3 or 4.0, the ethanol yields and enzyme efficiencies were significantly (*p* < 0.05) higher in fed-batch SScF (vertical vessel) at 20% (w/w) compared to batch SScF (horizontal vessel) at 30% (w/w) solid loading. However, for the ML substrate, only the *S. cerevisiae* strain Cellusec^®^ 4.0 produced significantly (*p* < 0.05) higher ethanol yields and enzyme efficiencies with fed-batch SScF.

The enzyme cost was stated to contribute 22% (on-site production) and 28% (off-site production) of the total cost of cellulosic ethanol [62], and in some cases 48% to the MESP [52] when produced off-site. The lowest enzyme cost of $0.19/L ethanol ($0.72/gal) was calculated for the high-solids fed-batch SScF process in the vertical bioreactor at 20% (w/w) solid loading fermenting LB with Cellusec^®^ 3.3. This was 48% lower than the enzyme cost of $0.31/L ($1.17/gal) calculated for the high-solids batch SScF process in the horizontal bioreactor at 20% (w/w) solid loading fermenting LB with Cellusec^®^ 3.3 (Fig. 8). Thus, the fed-batch SScF process in the vertical bioreactor is more favourable in terms of enzyme cost, than the high-solids batch SScF process in the horizontal bioreactor illustrated in Fig. 8. Compared to the enzyme cost of $0.34/gal obtained by Humbird et al., [53] that used on-site enzyme production, the $0.72/gal cost of enzymes in the current study was 2-fold higher, which may partly be attributed to off-site production [52, 53, 62] that was assumed in the current study. On the other hand, compared to $0.78/gal for off-site enzyme production determined in the study by Johnson [62] and $0.69 to $1.47/gal obtained by Klein-Marcuschamer et al., [77], the lowest cost of $0.72/gal obtained in the current study for the high-solids fed-batch SScF process compared well. This is a promising result for the possible industrial implementation of a high-solids fed-batch SScF waste paper to ethanol process.

## Conclusions

The study demonstrated the feasibility of using a horizontal bioreactor for high-solids batch SScF which was an unfamiliar approach for untreated LB, ML and PS paper wastes, producing ethanol titres up to 80.4 g/L at enzyme dosages $\:\le\:$ 5 FPU/g ds. Ultimately, high-solids fed-batch SScF in a vertical bioreactor at 20% (w/w) solid loading and 2 FPU/g ds was preferred to the high-solids batch SScF in a horizontal bioreactor, evident from the lower enzyme costs, higher enzyme efficiencies and ethanol yields of up to 0.109 g ethanol/FPU and 58.5% of theoretical maximum, respectively, this also indicates the potential for scale up. The lowest exogenous enzyme dosage of 2 FPU/g ds resulted in the highest enzyme efficiencies, regardless of the fermentation process, which could in part be due to substantial enzyme-production by the CBP yeast. It was determined that Cellusec^®^ 4.0 was the more viable CBP *S. cerevisiae* yeast strain, indicated by the performance stability, greater fermentation performance with the majority of the waste paper streams, and attractive thermotolerant properties. The findings from this study illustrate the attractive potential of unrecyclable waste paper as feedstock for ethanol production which would aid in diverting paper waste from landfill and incineration, and possibly incentivise the bioconversion of additional organic wastes.

## Supplementary Information

Below is the link to the electronic supplementary material.

Supplementary Material 1

## Data Availability

The raw data that support the findings of this study are available on request.

## Abbreviations

1G: First generation
2G: Second generation
ANOVA: Analysis of variance
CBP: Consolidated bioprocessing
CSL: Corn steep liquor
ds: Dry solids
FPU: Filter paper units
GHG: Greenhouse gas
LB: Label backing paper
ML: Multilayer paper-based packaging
MESP: Minimum ethanol selling price
PS: Potato paper sacks
SSF: Simultaneous saccharification and fermentation
SScF: Simultaneous saccharification and co-fermentation
YP: Yeast extract, peptone
YPD: Yeast extract, peptone, dextrose
