# Supplementary material for: High-solids ethanol production from waste paper through consolidated bioprocessing using advanced Saccharomyces cerevisiae strains
Source: Bioprocess Biosyst Eng. 2026 Aug 27;49(10):2793–810. doi: 10.1007/s00449-026-03416-5 (PMC13582411; doi:10.1007/s00449-026-03416-5)
Supplement: Supplementary file 1 [file 449_2026_3416_MOESM1_ESM.docx]

**High-solids ethanol production from waste paper through consolidated bioprocessing using advanced *Saccharomyces cerevisiae* strains**

**Bioprocess and Biosystems Engineering**

**Authors:** Juliana E. Naudé, Daneal C.S. Rorke*, Johann F. Görgens, Eugéne van Rensburg^#^

**Affiliations:** Dept. of Chemical Engineering, Stellenbosch University, Private Bag X1, Matieland, 7602, South Africa

*Corresponding author: Email: [rorked@sun.ac.za](mailto:rorked@sun.ac.za)

^#^Corresponding author - Eugene van Rensburg: Email – [eugenevrb@sun.ac.za](mailto:eugenevrb@sun.ac.za)

**Fermentation profiles comparing the effect of temperature on CBP *S. cerevisiae* Cellusec® 2.0, 3.3 and 4.0**

Fed-batch SScF (vertical vessel) at 20% (w/w) solid loading and an enzyme dosage of 2 FPU/g ds was conducted at temperatures of 37 ⁰C and 39 ⁰C with LB and PS substrates to investigate the effect of temperature on the *S. cerevisiae* CBP strains Cellusec® 2.0, 3.3 and the thermotolerant CBP strain Cellusec® 4.0. The performance of cellulase enzymes improve at increased temperatures [15], which would make the use of a thermotolerant CBP yeast strain more attractive.

**Fig.1S** Ethanol concentration (g/L) of PS (a) and LB (b) using Cellusec® 2.0 (37 ⁰C indicated by squares, 39 ⁰C indicated by x), Cellusec® 3.3 (37 ⁰C indicated by diamonds, 39 ⁰C indicated by triangles) and Cellusec® 4.0 (37 ⁰C indicated by circles, 39 ⁰C indicated by circles and dashed line) at 37 ⁰C and 39 ⁰C in high-solids fed-batch SScF at 20% (w/w) final dry solids loading and a Cellic® CTec3-HS dosage of 2 FPU/g ds for 144 h with CSL medium. Error bars represent the standard deviation of triplicate experiments. Arrows represent when 5% (w/w) dry solids were fed

The Cellusec® 4.0 strain achieved higher final ethanol concentrations at 39 ⁰C with the LB substrate than at 37 ⁰C, while the Cellusec® 2.0 and 3.3 strains preferred a fermentation temperature of 37 ⁰C, regardless of the waste-paper substrate (**Fig.1S**). Conversely, with PS as the substrate the performance of Cellusec® 4.0 was better at 37 ⁰C than 39 ⁰C, with no significant difference compared to Cellusec® 2.0 and 3.3. The improved performance of the thermotolerant Cellusec® 4.0 strain was attributed to the increase in cellulase activities at elevated temperatures [15]. Such thermotolerant properties are also likely to add robustness to industrial fermentation processes.

**Enzyme cost vs enzyme dosage for high-solids fed-batch SScF (vertical vessel) and high-solids batch SScF (horizontal vessel)**

**Fig.2S** Enzyme cost ($/L ethanol) for high-solids fed-batch SScF (vertical vessel) at 20% (w/w) final dry solids loading using Cellusec® 2.0, Cellusec® 3.3 and Cellusec® 4.0 and Cellic® CTec3-HS dosage of 2, 3.5 and 5 FPU/g ds at 120 h

**Fig.3S** Enzyme cost ($/L ethanol) for high-solids fed-batch SScF (vertical vessel) at 30% (w/w) final dry solids loading using Cellusec® 2.0, Cellusec® 3.3 and Cellusec® 4.0 and Cellic® CTec3-HS dosage of 2, 3.5 and 5 FPU/g ds at 120 h

**Fig.4S** Enzyme cost ($/L ethanol) for high-solids batch SScF (horizontal vessel) at 30% (w/w) final dry solids loading using Cellusec® 2.0, Cellusec® 3.3 and Cellusec® 4.0 and Cellic® CTec3-HS dosage of 2, 3.5 and 5 FPU/g ds at 120 h

**Fig.5S** Enzyme cost ($/L ethanol) for high-solids batch SScF (horizontal vessel) at 40% (w/w) final dry solids loading using Cellusec® 2.0, Cellusec® 3.3 and Cellusec® 4.0 and Cellic® CTec3-HS dosage of 2, 3.5 and 5 FPU/g ds at 120 h
